# Effects of Selection for Honey Bee Worker Reproduction on Foraging Traits

**DOI:** 10.1371/journal.pbio.0060056

**Published:** 2008-03-04

**Authors:** Benjamin P Oldroyd, Madeleine Beekman

**Affiliations:** Behaviour and Genetics of Social Insects Laboratory, School of Biological Sciences A12, University of Sydney, New South Wales, Australia; University of Lausanne, Switzerland

## Abstract

The “reproductive ground plan” hypothesis (RGPH) proposes that reproductive division of labour in social insects had its antecedents in the ancient gene regulatory networks that evolved to regulate the foraging and reproductive phases of their solitary ancestors. Thus, queens express traits that are characteristic of the reproductive phase of solitary insects, whereas workers express traits characteristic of the foraging phase. The RGPH has also been extended to help understand the regulation of age polyethism within the worker caste and more recently to explain differences in the foraging specialisations of individual honey bee workers. Foragers that specialise in collecting proteinaceous pollen are hypothesised to have higher reproductive potential than individuals that preferentially forage for nectar because genes that were ancestrally associated with the reproductive phase are active. We investigated the links between honey bee worker foraging behaviour and reproductive traits by comparing the foraging preferences of a line of workers that has been selected for high rates of worker reproduction with the preferences of wild-type bees. We show that while selection for reproductive behaviour in workers has not altered foraging preferences, the age at onset of foraging of our selected line has been increased. Our findings therefore support the hypothesis that age polyethism is related to the reproductive ground plan, but they cast doubt on recent suggestions that foraging preferences and reproductive traits are pleiotropically linked.

## Author Summary

In social insects, the evolution of the worker caste and the regulation of reproductive behaviour by workers are poorly understood. Evolution is conservative and often proceeds by adapting an existing gene network to a new function. The “reproductive ground plan” hypothesis (RGPH) suggests that social insects evolved their queen and worker castes by modifying a gene network that once regulated the foraging and reproductive phases of solitary ancestors. In this model, queens retain characteristics of insects in their reproductive phase, whereas workers retain characteristics of the foraging phase. Moreover, the foraging behaviour of workers may also be regulated by the same genes that once controlled the switch between foraging and feeding young in the nest. We evaluated the RGPH by studying a line of honey bees selected for high rates of worker reproduction. We show that in this line workers forage late in life and some may never forage, supporting the idea that genes related to reproduction are also related to foraging. However, we found no support for recent suggestions that genes related to reproduction also regulate the foraging behaviour of individual workers: once they start foraging, our highly reproductive workers forage in the same way that unselected workers do.

## Abstract 3

A new study lends support to the model of social insect evolution, in which a gene regulatory network linked to the reproductive and foraging phases of ancestral solitary insects gave rise to queen and worker castes. But the study challenges the notion that genes related to reproduction also regulate the foraging behavior of workers.

## Introduction

Two of the most challenging questions to students of social insects are the evolutionary origins of the worker caste [e.g., 1–6] and the regulation of division of labour within the worker caste [e.g., 7–13]. It has been suggested that the solution to both these puzzles may lie in modifications of the basal reproductive cycle of solitary insect species. West-Eberhard [14] and Gadagkar [15] have argued that in social species, queens and workers have lost different parts of the original reproductive cycle of solitary species. In some solitary insects, females oscillate between an oviposition phase when ovaries are active, and a foraging phase where the female gathers food for her developing larva(e). This oscillation is regulated by cycling levels of juvenile hormone, ecdysteroids, and vitellogenin [16,17]. During the reproductive phase, ovaries are active and titres of circulating vitellogenin—the egg protein precursor—are high. During the foraging phase, the insect's ovaries are nonactive, and circulating vitellogenin titres are low. After her larvae have begun to pupate, the adult solitary female may revert to a new ovulatory phase where her ovaries become reactivated and foraging is curtailed. West-Eberhard and Gadagkar propose that in some social wasps like *Polistes*, workers express only the foraging phase and the reproductive phase has been lost, whereas in queens, the reproductive phase is expressed and the foraging phase has been lost. We call this the original “reproductive ground plan” hypothesis (original RGPH, Table 1). Hunt and Amdam [18] proposed a modification of this idea, arguing that in *Polistes* wasps at least, queens and workers arise from two developmental pathways that characterise the bivoltine lifecycle of some solitary species (bivoltine RGPH, Table 1). They suggest that queens evolved via co-option of the gene regulatory networks that are switched on in late-emerging, second-generation individuals that diapause, and the worker caste from networks switched on in early-emerging first generation individuals that do not enter diapause.

**Table 1:**
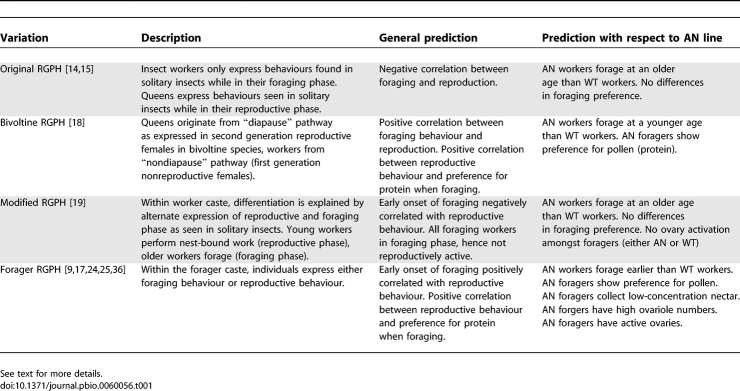
Summary of the Different Variations of the RGPH, Their General Predictions with Respect to the Correlation between Foraging Behavior and Reproduction, and Our Specific Predictions with Respect to the Selected AN Line

West-Eberhard [19] extended her original RGPH [14] from being solely an explanation of the evolution of caste by suggesting that the changes in behavioural phenotype that typically occur as social insect workers mature may also have their antecedents in the reproductive ground plan of their nonsocial ancestors, i.e., between the reproductive and nonreproductive phases of a solitary female's adult life. This idea stems from the fact that in some social species, young nest-bound workers retain characteristics of solitary females in their reproductive phase: some ovary activation, aggressiveness towards intruders, and in-nest work, rather than foraging. Later in life, workers cease larval feeding and engage in foraging, retaining some features of the nonreproductive phase, such as inactive ovaries. This idea has found support in the honey bee, Apis mellifera, where workers perform nest-bound tasks early in life and start foraging when older [20–22]. Furthermore, young honey bee workers that are engaged in brood care have high levels of circulating vitellogenin and may have some thickening of their ovaries. Older individuals engaged in foraging have low vitellogenin titres, and their ovaries are completely regressed [23]. We call this the modified RGPH (Table 1).

The most recent version of the RGPH argues that in honey bees, division of labour within the *foraging* population (rather than between queen and worker castes or between nursing and foraging workers) also has its antecedents in the gene regulatory network that once regulated the gonotrophic cycle in solitary ancestors of the social bees [9,17,24,25]. Within a honey bee colony's population of foragers, there is variability among individuals for foraging preferences, and this specialisation has a genetic component (reviewed in [7,12,13]). In particular, some foragers specialise in collecting pollen, whereas others specialise in collecting water or nectar. It is argued that the basal cycles of reproduction and foraging now regulate division of labour between pollen and nectar foragers and drive foraging behaviour [17]. Especially important here are the genes that regulate a bee's degree of attraction to the concentration of sugar in nectar, and the age at which workers make the transition from in-nest tasks to foraging and other tasks external to the nest [17,26,27]. We refer to this as the forager RGPH (Table 1).

Experimental support for the explanatory power of the forager RGPH in the evolution of foraging specialisation in the honey bee has come from studies of two lines selected by R.E. Page, Jr. and colleagues for high and low pollen hoarding [25,28,29]. Relative to workers from the low–pollen hoarding line and to unselected workers, workers from the high–pollen hoarding line start foraging early in life, carry pollen more frequently, carry larger pollen loads, have more ovarioles, are more responsive to low concentrations of sucrose, have elevated levels of vitellogenin, and are more likely to show pre-vitellogenic swelling of their ovaries [24,25,28,30–35]. This divergence between the behaviour and reproductive physiology of the two selected lines has been interpreted within the conceptual framework of the forager RGPH as a demonstration of how the “behavioral mechanisms of division of labour evolve from solitary ancestry, and provides an experimental demonstration of the origins of sib-care behavior from maternal reproductive traits” [24]. Thus correlations between reproductive characters such as ovariole number and levels of ovary activation—however slight—with components of foraging behaviour such as preference for pollen over nectar and the age at which foraging commences, are interpreted as showing that division of labour in foraging is mediated pleiotropically by the same gene networks and hormonal cascades that mediate reproductive behaviour in workers [17,24,25,36]. Thus it is argued [17,24] that selection for high pollen hoarding has resulted in a line that displays characteristics of the reproductive phase of the solitary insect's life cycle in which they actively seek protein. In contrast, the low–pollen hoarding line is thought to show characteristics that reflect solitary insects in their nonreproductive phase where they seek carbohydrate.

We investigated the link between reproduction and reproductive division of labour by studying the foraging behavior of a line of “anarchistic” (AN) honey bees that has been selected for worker reproduction and in which about 1/3 of 10-d-old queenright workers show activation of their ovaries and the presence of oocytes in their ovarioles [37–40]. We evaluated the predictions of the various versions of the RGPH (Table 1) by a comparison of the foraging behaviour and reproductive physiology of workers of this line with that of unselected wild-type (WT) workers. We found experimental support for the modified RGPH but no support for the forager extension of the original hypothesis.

## Materials and Methods

We performed our experiment in duplicate, once in January 2007 and once in November 2007. For each replicate, we chose two WT colonies of standard Australian commercial stock (primarily *Apis mellifera ligustica* in origin) headed by open-mated queens and two AN colonies from the line maintained at the University of Sydney via artificial insemination [37,40]. Workers were confirmed to be reproductively active in the AN colonies by the presence of drone brood laid by workers. We confined the queen in each of the four colonies on an empty brood comb by means of a cage constructed of queen excluder material that allows workers to pass through, but not the larger queens [41]. The queens were confined to the combs for 2 d, after which we transferred the four egg-laden combs into a single WT colony in a part of the nest in which the host queen could not enter because of a queen excluder. The genotype of the rearing colony is known to have minimal effect on the expression of anarchistic traits [38], but we used uniform rearing environments to minimise any possible rearing effects.

The host colonies reared the eggs to the late pupal stage, whereupon we transferred the brood combs into an incubator at 34.5 °C and high relative humidity. Over 2 d, we marked 1,000 emerging workers from each of the four source colonies with coloured paints (Posca Posta Pens, Japan) on their thorax to identify each worker as to its colony of origin and day of emergence.

For each replicate, we transferred the 4,000 marked workers into a single, WT colony, unrelated to any of the source colonies nor the rearing colony, comprising six combs of adult bees and brood, and moved the host colony to Pearl Beach, NSW, Australia, for study. During both replicates, pollen and nectar were abundantly available from a variety of native and introduced species, especially Angophora floribunda in January, and *An. hispida* during November.

In both replicates, some marked bees were seen at the entrance to the host colony 5 d after introduction, and we commenced collecting all marked workers that returned to the colony beginning on day 6 to determine the age at which workers of each source commenced foraging. To do this, we reduced the width of the entrance of the nest to 2 cm with a block of wood. Using forceps, we grasped all paint-marked returning foragers and placed them individually in microcentrifuge tubes. We placed the tubes containing the workers on a frozen “blue ice” brick in a polystyrene box to cool them to immobility. Except when foraging was interrupted by rain, we collected foragers from 09:00 to 13:00, until we had collected approximately the first 100 bees to forage from each source colony. We avoided collecting foragers in the afternoon when workers make their first orientation flights but do not forage at flowers [42].

The day at which the first foraging workers were collected was used as a measure of age at first foraging. This estimate is skewed to the earliest foragers within each group, and is not an average age at first foraging. However, we wished to leave workers to mature further so that we could obtain an unbiased estimate of their foraging preference – for nectar or for pollen. We preferred to use experienced foragers, because we saw almost no pollen in our samples of first-foraging bees (a few workers carried traces of pollen). This was despite the fact that host colony foragers collected large pollen loads.

When the workers were 21 d old, we commenced collecting a second set of workers. Most workers are foragers by this age [30,42–44]. We used these workers to determine the foraging preference for nectar and pollen of AN and WT workers. Again we collected returning foragers between 09:00 and 13:00. For each replicate, we collected ten workers derived from each source colony on each of 4 d. After collecting the final set of foragers, we moved the host colony 10 m away from its original location and replaced it with a dummy hive containing a comb of sealed brood. Any experienced foragers that left the colony to forage returned to the dummy hive at the original location. After the host colony had been moved aside for 6 h, we opened it and collected all of the remaining marked bees. We assume that most of the bees in this sample had never foraged.

For the sample of foragers collected at age 21–26 d (on some days, we could not collect foragers because of rain), we determined for each bee: (1) the volume of nectar she carried in her crop; (2) the concentration of sugar in the solution carried (if any was present), and (3) the mass of any pollen she carried.

To retrieve nectar from the crop of each bee, we gently squeezed her abdomen between thumb and forefinger and caused her to regurgitate the contents of her crop. We drew the contents of the crop into a 50-μl microcapillary tube (Drummond Scientific) and then measured the length of the liquid column with a ruler. We then converted this length into a volume in μl [45]. Where more than 2–3 μl were retrieved, it was generally possible to obtain a measure of the mass of dissolved sugars in the regurgitated crop contents using a hand-held refractometer with a range of 0–80 brix (Meopta, Taiwan).

To determine the mass of the pollen loads, we scraped the pellet off the bee's basitarsi into a pre-weighed microcentrifuge tube. We then weighed the tube and pellet and deducted the mass of the tube. Where only one pellet was retrieved, we doubled the mass of the single pellet.

To understand the effects of ovary activation and number of ovarioles on worker foraging behaviour, we dissected two groups of workers as follows: (1) All 160 experienced foragers (i.e., aged 21–26 d old, 40 from each source colony). This sample allowed us to explore relationships between pollen and nectar foraging preference, degree of ovary activation, and ovariole number. (2) A matching sample of 160 “non”-forager workers, 40 from each source colony, caught from inside the host colony after this had been moved aside, causing foragers to return to the dummy hive. These dissections allowed us to explore how late onset of foraging (or lack of foraging) was associated with ovary activation and ovariole number.

To dissect workers, we pinned them to a wax plate through the thorax. We then pulled the abdomen apart using fine forceps between tergites 4 and 5 while irrigating with water. We retrieved the ovaries, scored them on a scale of 0–4 for signs of ovary activation, and counted the number of ovarioles [24,38].

## Results

### Genotypic Differences in Age at First Foraging

**Figure 1:**
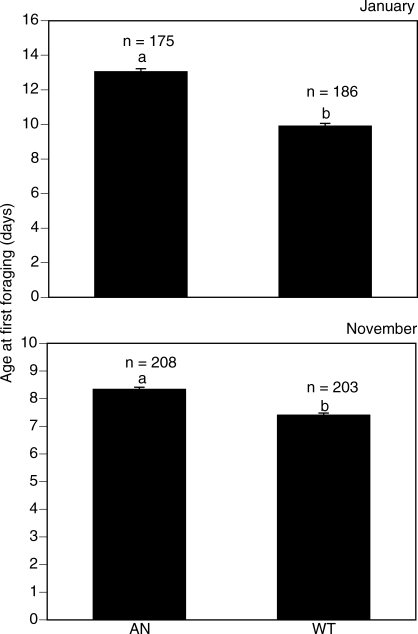
Mean Age at Which the First Workers to Forage Returned from Their First Foraging Trip Within each replicate (January and November), means followed by a different letter are significantly different at the 5% level (based on least significant differences from univariate ANOVA). Bars are standard errors of the means. AN = anarchist colonies; WT = wild-type colonies; *n* = the sample size.

Because of a significant genotype x replicate interaction (*F*_1,768_ = 99.3, *p* < 0.001, two-factor ANOVA with fixed effects) caused by a significant effect of replicate (*p* < 0.001), we were unable to pool data from the two replicates. However in both replicates, AN workers were significantly older than WT workers on their first foraging flight. In the January replicate, the first workers to forage from the two AN colonies were on average 13.4 d old, whereas the WT workers commenced foraging significantly earlier (*p* < 0.001, two-tailed *t*-test) when 9.9 d old (Figure 1). In the November replicate, the first AN workers to forage were 8.3 d old. Again, WT workers commenced foraging significantly (*p* < 0.001) earlier when 7.3 d old. Furthermore, of the 439 marked workers present in the replicate 1 colony at the end of the experiment, which most likely had never foraged, 313 were AN and 126 were WT. Similarly in the November replicate, there were 417 workers remaining of which 279 were AN and 138 were WT. Because equal numbers (2,000 each) of AN and WT workers were introduced to each of the host colonies, this is suggestive that a larger proportion of mature AN workers refrained from foraging than did WT workers. Under a hypothesis that the number of workers of each genotype expected to not engage in foraging should be equal, there were significantly more AN workers present in the nonforaging populations than there were WT workers (January, 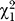 = 79.66, *p* < 0.001; November, 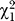 = 47.68, *p* < 0.001).

### Foraging Preferences of Mature Foragers

**Figure 2:**
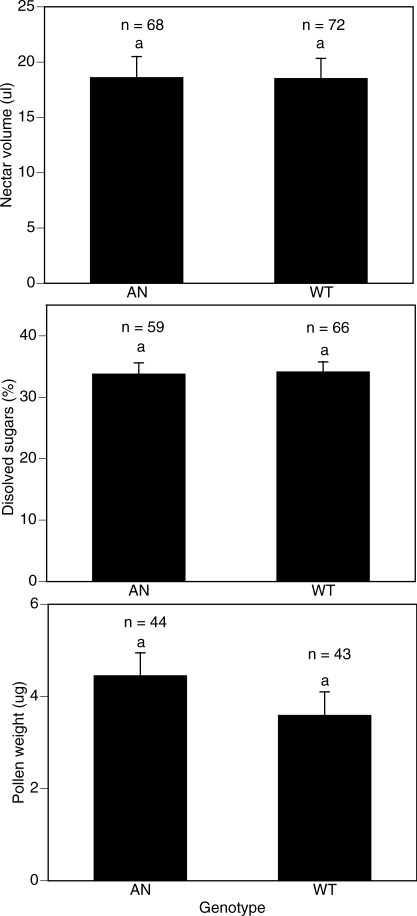
Loads Carried by Mature (>21 d Old) AN and WT Foragers Means followed by the same letter are not significantly different (two-way ANOVAs of genotype and replicate). Bars are the standard errors of the means. *n* = the sample size.

There were no significant genotype (AN versus WT) by replicate (January versus November) interactions (ANOVA using fixed effects models) for the three variables measured on mature foragers: nectar volume (*F*_1,136_ = 0.58, *p* = 0.45, excluding workers that carried no nectar), nectar concentration (*F*_1,121_ = 0.008, *p* = 0.93*,* excluding workers that carried no nectar) and weight of pollen carried (*F*_1,83_ = 1.42, *p* = 0.24, excluding workers that carried no pollen). We therefore analysed data as two-factor ANOVAs of genotype and replicate, in which we tested whether AN and WT workers differed in the kinds of forage they collected. There was no significant difference in the foraging preferences of AN or WT workers for any parameter measured (Figure 2). There were significant replicate effects for nectar volume (*F*_1,136_ = 9.30, *p* = 0.003), nectar concentration (*F*_1,121_ = 6.19, *p* < 0.014), and weight of pollen carried (*F*_1,83_ = 125.28, *p* < 0.001).

### Ovary Development and Activation in Foraging and Nonforaging Workers

**Figure 3:**
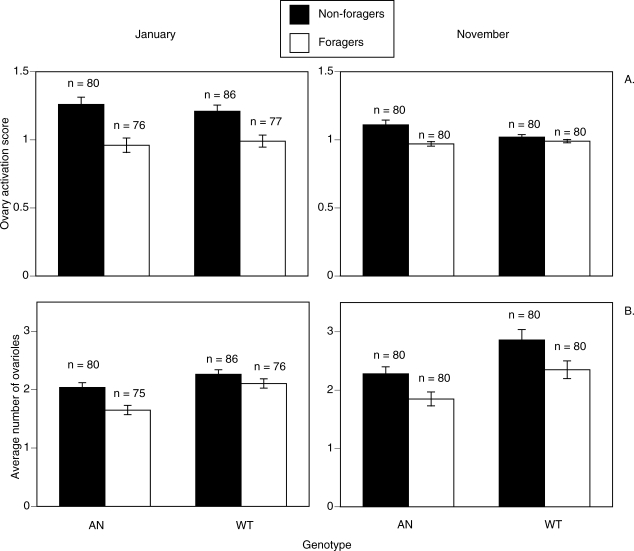
Ovary Activation and Number of Ovarioles in Mature (>21 d Old) Workers of WT and AN Source Colonies That Were Either Active Foragers or Which Had Probably Never Foraged (A) Ovary activation score. Ovary activation was scored in the range 0–4. Bars indicate the standard errors of the means. *n* = the sample size. (B) Average number of ovarioles. This is the average number of ovarioles in the left and right ovary. Where only one ovary was visible, that ovary alone was used.

In both replicates, mature AN workers that had probably never foraged had a significantly higher number of ovarioles than AN individuals of similar age that were collected after foraging (January, Mann-Whitney *U* = 2,236.5, *p* = 0.005, November, *U* = 2,385.5, *p* = 0.005) (Figure 3). There was no significant difference between the number of ovarioles in foraging and nonforaging WT workers in January (*U* = 3,002.5, *p* = 0.4) but there was in November (*U* = 2,545.0, *p* = 0.02). WT workers had significantly more ovarioles than did AN workers in both the forager group (January, *U* = 1,949.5 *p* < 0.001; November, *U* = 2,442.0, *p* = 0.009) and among nonforagers (January, *U* = 2,900.0, *p* = 0.07, November, *U* = 2533.5, *p* = 0.02) (Figure 3). For AN workers, the nonforaging group had higher ovary activation scores than the foragers (January, *U* = 2,209.0, *p* < 0.001; November, *U* = 2,769.0, *p* < 0.001) (Figure 3) and immature ova (ovary activation score 3) were observed in a single nonforaging AN worker in January. For WT workers, the nonforaging group had significantly higher activation scores than foragers in January (*U* = 2,602.0, *p* < 0.001) but not in November (*U* = 3,081.0, *p* = 0.84) (Figure 3). There was a positive correlation between ovary activation score and average number of ovaries among all non-foraging workers (Spearman correlation, ρ = 0.135, *n* = 326, *p* = 0.01).

### Relationships between Ovary Development, Ovary Activation, and Foraging Behaviour

There was no significant association between genotype and whether a worker carried pollen (January 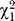 = 0.03, *p* = 0.85; November, 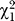 = 0.64, *p* = 0.42). The number of ovarioles was uncorrelated with the mass of pollen carried (*r* = 0.13, *n* = 127, *p* = 0.88) or the volume (*r* = −0.094, *n* = 311, *p* = 0.10) or concentration (*r* = 0.07, *n* = 158, *p =* 0.35) of nectar carried. (These correlations were also not significant on a per replicate basis.) Ovary activation scores were significantly correlated with nectar concentration (Spearman's ρ *=* 0.25, *n* = 159, *p* = 0.002), but not with nectar volume (ρ *=* 0.08, *n* = 313, *p* = 0.14) or pollen mass (ρ *=* −0.051, *n* = 127, *p* = 0. 60). There was a significant correlation between the number of ovarioles and ovary activation scores (ρ = 0.29, *n* = 311, *p* < 0.001). These patterns of significance were identical on a per replicate basis.

## Discussion

Our data show that AN workers forage significantly later in life than WT workers, and that AN and WT workers were equally likely to forage for nectar or pollen, foraged for nectar of similar quality, and carried similar-sized pollen loads. Thus, our data support the modified RGPH, which suggests that workers selected for high reproductive rate should have late onset of foraging, but otherwise should not differ in foraging behavior. Our data do not support the forager RGPH, which predicts that AN workers should commence foraging early in life and focus on foraging for proteinaceous pollen (Table 1) [8,25,36].

We found a positive association between ovary activation and ovariole number. This suggests that a larger number of ovarioles, laid down in the larval stage, increases the probability that the individual will become reproductively active. These findings accord with findings in other honey bee populations [24,46,47]. There was a positive correlation between ovary activation scores and nectar concentration. We have no explanation for this, but we note that it is contrary to a prediction of the forager RGPH hypothesis, which posits that more reproductive workers should forage for pollen and nectar of low sugar concentration.

Independent support for a link between the tendency of honey bee workers to delay or refrain from foraging and their reproductive potential comes from the Cape honey bee (*A. m. capensis*) of South Africa. Uniquely, workers of this subspecies are thelytokous and therefore produce female offspring from their unfertilised eggs [48–50]. Because of this, natural selection strongly favours worker reproduction, because workers have the opportunity to contribute directly to the pool of eggs that are raised as queens [51–54]. Consistent with the modified RGPH (Table 1) [19], it is easy to identify two distinct kinds of workers in *A. m. capensis*. Dominant workers do little work but express traits that are indicative of high reproductive potential. Subordinate workers, by contrast, do the majority of the work, but are reproductively inactive [55,56].

The observation that workers of a strain selected for high pollen hoarding show increased vitellogenin titres relative to a strain selected for low pollen hoarding has provided important support for the forager RGPH hypothesis [23,36]. The level of circulating vitellogenin is a good predictor of reproductive potential in many social insects (e.g., [57,58]). It thus seems logical to postulate a role for vitellogenin in the regulation of reproductive potential in honey bee workers, and that workers with high vitellogenin titres should have higher reproductive potential than those that have low titres [36]. However, nurse workers need to produce large amounts of vitellogenin in order to produce brood food that is fed to larvae [23]. Once workers have commenced foraging, they no longer need to produce brood food, and vitellogenin titres are reduced while juvenile hormone titres increase [59]. We therefore think that vitellogenin titres of honey bee workers, contrary to many other social insects, may not be a reliable predictor of an individual's direct reproductive potential.

We also doubt the validity of a general association between reproductive potential and division of labour when foraging, modulated by the production of vitellogenin. Solitary bees like Megachilidae actively forage for pollen and nectar, building and provisioning brood cells, one at the time. Once a cell has been provisioned, the female oviposits on the pollen mass and commences foraging to provision another cell. In these species, there is no nest-bound phase for adults, and foraging and reproductive behaviours are contemporaneous. If honey bees evolved from an ancestor similar to the Megachilidae, then it seems unlikely that the gene networks that regulate alternate life history phases would also regulate foraging. Alternatively, the honey bee's ancestor could have had a life cycle similar to Xylocopini and Ceratinini bees, where a nonreproductive female (daughter or unrelated female) remains inside the nest and guards it [60]. In these species, it is the reproductive female who does the foraging and egg laying, the nonreproductive, nest-bound female merely waits to inherit the nest. If honey bees evolved from this kind of bee, then one would predict that nest-bound workers would have the lowest reproductive potential in accordance with our results as well as the modified RGPH.

Our study highlights the pitfalls of making general conclusions about the evolution of behaviour from particular selected lines when the underlying genetic mechanisms behind behavior are poorly understood. Page et al.'s line was selected for pollen hoarding and this has affected some reproductive traits and age at first foraging. Our line was selected for reproduction, and this had no effect on foraging preferences and has increased age at first foraging. The divergent results when selecting on different phenotypes is explainable by weak genetic correlations between the traits in question. Thus selection on one trait (pollen hoarding) selects on a different component of variation related to onset of foraging and reproductive potential than direct selection for worker reproduction. Hence, the observed correlation between the tendency to forage for pollen and early onset of foraging can simply be an artefact of selection on gene networks unrelated to reproductive potential.

## Abbreviations

AN: anarchistic
RGPH: reproductive ground plan hypothesis
SE: standard error
WT: wild type
